# Non-thyroidal illness (euthyroid sick) syndrome: Laboratory aspects and clinical significance in critically ill patients and other diseases – A narrative review

**DOI:** 10.2478/jccm-2026-0008

**Published:** 2026-01-30

**Authors:** Liong Boy Kurniawan

**Affiliations:** Department of Clinical Pathology, Faculty of Medicine, Hasanuddin University, Makassar, Indonesia

**Keywords:** NTIS, critical illness, infectious diseases, non-infectious diseases

## Abstract

Formerly termed euthyroid sick syndrome, non-thyroidal sickness syndrome (NTIS) is a disorder that frequently occurs in acute or chronic illnesses that alter the levels of thyroid hormone and patterns, even in the absence of hypothalamic-pituitary-thyroid axis problems or diseases. The primary findings on the thyroid hormone panel in NTIS are elevated reverse T3 (rT3) and decreased triiodothyronine (T3) levels, which may be followed by other thyroid hormone abnormalities, such as thyroid-stimulating hormone (TSH) and thyroxine (T4). The incidence of NTIS increases among hospitalized patients with critical illness, and there is an associated increase in mortality. NTIS is also associated with worsening outcomes during and after treatment in patients hospitalized with infectious or non-infectious diseases, such as cardiovascular, kidney, lung, diabetes mellitus, autoimmune, and other diseases. In patients with critical illnesses admitted to the Intensive Care Unit (ICU), serial examination of a panel of thyroid function tests, including T3 and rT3, is necessary to estimate the phase of the disease (whether acute, chronic, or recovery) and can be used to predict the risk of mortality during treatment.

## Introduction

One of the most common laboratory tests in the endocrine field is the thyroid function test. Thyroid function investigations are routinely conducted in instances of suspected hyperthyroidism or hypothyroidism, whether presenting clinically/subclinically [1]. These evaluations are conducted across the lifespan, encompassing congenital hypothyroidism neonatal screening and the confirmation of diagnoses related to thyroid dysfunction, particularly Hashimoto’s thyroiditis and Graves’ disease, which are prevalent among adults, and other thyroid disorders [1,2].

Thyroid function tests are utilized to evaluate the axis of hypothalamic-pituitary-thyroid. Thyrotropin-releasing hormone (TRH) is synthesized by the hypothalamic paraventricular nucleus. The anterior pituitary then produces thyrotropin/thyroid-stimulating hormone (TSH) in response to TRH. Subsequently, TSH will trigger thyroid gland to produce triiodothyronine (T3) and thyroxine/tetraiodothyronine (T4). T4 is the main thyroid hormone released by the thyroid gland, accounting for around 80% of the total production. The remaining 20% is T3. As indicated by the extant literature, blood levels of T4 and T3 have been demonstrated to affect the feedback production of hypothalamic TRH and pituitary TSH [3,4,5,6].

A variety of acute and chronic diseases have the potential to affect the axis of hypothalamus-pituitary-thyroid, which can result in low blood T3 levels. This condition was previously known as euthyroid sick syndrome, as the patient was still considered euthyroid despite the presence of low blood T3 levels. In the present era, the term “NTIS” has become increasingly predominant in scientific discourse, superseding the previously dominant term, “euthyroid sick syndrome.” The “NTIS” term is used to define acute or chronic/prolonged disease conditions that result in alterations in the levels and patterns of thyroid hormone, despite the absence of an intrinsic disorder or disease in the axis of hypothalamic-pituitary-thyroid [7,8,9]. Early detection of NTIS in critically ill hospitalized patients is crucial because it is strongly associated with disease severity, prognosis, and patient mortality.

In this narrative review, we explore the laboratory aspects of NTIS and its clinical significance in critically ill patients and other diseases. For this, we conducted an online literature search in the PubMed and Google databases. The keywords used in the literature search were “non-thyroidal illness” OR “NTI” OR “NTIS” OR “euthyroid sick syndrome”, AND “laboratory aspects” OR “laboratory tests”, AND “clinical significance”, AND “critically ill patients”, AND “other diseases”. The literature/studies collected were from the period 2002 to 2024.

## NTIS and outcomes among critically ill patients hospitalized in the intensive care unit (ICU)

The relationship between alteration in the thyroid hormone patterns, the prevalence of NTIS, outcomes and survival of patients with critical illness hospitalized in the ICU has been an interesting subject of study. Wang et al. performed a study of 480 ICU patients in a Shanghai, China hospital and found that mean T3, T4, free T3 (FT3), free T4 (FT4), and TSH levels were lower in non-surviving subjects than in surviving ones (T3 = 0.89±0.30 vs. 1.16±0.32 nmol/L; T4 = 59.52±21.92 vs. 73.92±20.88 nmol/L; FT3 = 2.95±0.57 vs. 3.53±0.60 pmol/L; FT4 = 14.48±3.66 vs. 15.80±3.29 pmol/L; median TSH = 0.60 vs. 0.87 IU/mL; all p<0.05). Compared to other thyroid hormone parameters, FT3 had the greatest area under the curve (AUC) value in predicting mortality in critically ill patients (cut-off<3.33 pmol/L, AUC = 0.762, specificity = 0.78, sensitivity = 0.62) [10].

Praven et al. conducted a study of 119 critically ill adult patients in Hyderabad, India, and found that the incidence of NTIS was lower among survivors than non-survivors (63% vs. 93%). Among thyroid hormone parameters, blood levels of T3 and TSH were lower among non-survivors compared to survivors (T3 = 0.46±0.20 vs. 0.58±0.22 ng/dL; TSH = 1.75±2.27 vs. 2.23±1.66 IU/mL; all p<0.05). Compared to other thyroid parameters, T3 had the highest AUC value in predicting mortality (AUC = 0.677, specificity = 0.56, sensitivity = 0.83) [11].

Similar reports have been documented in critically ill pediatric patients undergoing treatment. Sayarifard et al. (Tehran, Iran) conducted a study on children with critical illness hospitalized in the pediatric intensive care unit (PICU) to assess the association between thyroid hormones and survival. T3 levels on day 7 were lower in patients who did not survive than in those who did (76.70±18.85 vs. 100.46±27.60 ng/dL, p<0.05), as were T4 levels on day 3 (3.94±2.86 vs. 7.56±3.34 μg/dL, p<0.05). Surviving patients showed increased blood levels of T3, T4, FT3, and FT4 on day 3 compared to day 1, while non-surviving patients showed decreased T3, T4, FT3, and FT4 levels on day 3 compared to day 1 [12]. Carreras et al. (Spain) conducted a study of pediatric patients admitted to the PICU. Patients at high risk of mortality, as assessed by the Pediatric Risk of Mortality (PRISM) III score, had significantly lower TSH and FT4 levels compared with patients at low risk of mortality. An FT4 level below 16.60 pmol/L can be used to determine an increased risk of mortality (AUC = 0.655, specificity = 0.61, sensitivity = 0.76). Patients with NTIS had an increased mortality risk 6.04 times higher than non-NTIS subjects, and those with FT4 levels below 16.6 pmol/L had an increased risk of mortality 5.15 times higher than those with FT4 levels above 16.6 pmol/L [13].

Another study of ICU patients with critical illness in Wuxi, China, reported a correlation between levels of serum rT3 and patient’s condition severity (as assessed by the score of Acute Physiology and Chronic Health Evaluation II [APACHE II]): the higher the score of APACHE II, the higher the serum reverse T3 (rT3) levels (r = 0.379, p = 0.004). The score of APACHE II was negatively correlated with blood levels of TSH, T3, and T4, suggesting the suppression of TSH production and the reduced binding of serum T3 and T4 by transporter proteins as the disease progressed [14]. In contrast, Krug et al. performed a study of 1,790 adult ICU patients and found that NTIS was not associated with mortality increased risk. However, NTIS was correlated with an elevated risk of ICU stay (odds ratio [OR] = 1.023), an increased need for ventilator use (OR = 2.022), and an increased incidence of liver failure (OR = 2.823) [15].

Vidart et al. conducted a meta-analysis including 6,869 ICU patients with critical illness. Based to the findings, NTIS was a predictor that was independently linked to a higher risk of death (OR = 2.21). Although there was no discernible difference in TSH levels between the two groups, the non-survivor group had lower levels of T3, FT3, and FT4 than the survivor group [16].

The association between thyroid hormones, NTIS, and outcomes in critically ill patients is compiled in Table 1.

**Table 1.** The relationship between NTIS, thyroid hormones and outcomes in critically ill patients

**Authors**	**Country**	**Subjects**	**Age**	**Main Findings**
Wang et al.[10]	Shanghai, China	480 ICU patients	71.71 years (mean)	T3, T4, FT3, FT4, TSH levels were lower in non-survive compare to survive patients
				FT3 (cut-off <3.33 pmol/L) has the largest AUC (0.762) to predict mortality (specificity = 0.78, sensitivity = 0.62)

Praven et al.[11]	Hyderabad, India	119 critically ill patients	60.15 years (mean)	NTI prevalence was lower in survive (63%) compared to non-survive (93%) patients
				T3 and TSH levels were lower in non-survive compare to survive patients
				T3 has the largest AUC (0.677) to predict mortality (specificity = 0.56, sensitivity = 0.83)

Sayarifard et al.[12]	Tehran, Iran	35 children with critical illness	4 months to 15 years	T3 levels were lower among non-survive patients in the 1st day of hospitalization while T4 levels were lower in the 3rd day

Carreras et al.[13]	Spain	103 PICU patients	median 8.51 years (higher mortality risk score group) and 5.78 years (lower mortality risk score group)	FT4 (cut-off <16.60 pmol/L) has the largest AUC (0.655) to predict higher mortality risk (specificity = 0.615, sensitivity = 0.76)
				Subjects with NTIS had 6.04 times higher mortality risk while those with FT4<16.60 pmol/L had 4.92 times higher mortality risk

Wang et al.[14]	Wuxi, China	51 ICU patients	15 to 88 years	APACHE II score has positive correlation with rT3 (r = 0.379) and negative correlation with TSH (r =−0.256), T3 (r =−0.370), T4 (r =−0.364) (all p≤0.05)

Krug et al.[15]	Leipzig, German	1790 ICU patients	44–77 years (interquartile range)	TSH, FT3, and FT4 levels were lower in NTIS compared to non-NTIS
				NTIS condition was associated with longer ICU length of stay but not associated with higher ICU mortality rate

Vidart et al.[16]		6869 adult with critical illness (meta-analysis of 25 studies)		Subjects with NTIS had 2.21 times higher mortality risk compared to non-NTIS

## NTIS and its relationship with outcomes in infectious diseases and sepsis

Yao et al. conducted a study of 1,219 hospitalized sepsis patients in Shanghai, China, and found that NTIS was associated with the incidence of disseminated intravascular coagulation (DIC). NTIS contributes to the occurrence of DIC. Patients with sepsis and NTIS were 3.19 times more likely to have DIC than patients without NTIS. The higher the DIC score (measured according to the criteria of International Society on Thrombosis and Hemostasis (ISTH)), the higher the prevalence of NTIS. Subjects with DIC scores of 7–8 had a higher NTIS prevalence than those with scores of 5–6, 3–4, or 0–2 (76.3%, 55.7%, 36.1%, and 15.4%, respectively). The risk of mortality for sepsis patients with DIC throughout the 28-day monitoring period was 1.18 times higher for individuals with NTIS than for those without NTIS [17].

Abdelgawad et al. conducted a study in Cairo, Egypt, examining 40 sepsis/septic shock pediatric patients. On the first day of therapy, survivors had greater FT3 levels than non-survivors, and on the first and fifth days of treatment, survivors had lower rT3 levels than non-survivors. NTIS was more prevalent among non-surviving subjects (61.9%) than survivors (31.6%) [18].

Sharma et al. in India, who conducted a study on neonates with sepsis, reported similar results. Subjects with sepsis had considerably lower FT4 and FT3 levels than the control group, but there was no discernible change in TSH levels. Compared to surviving individuals, non-surviving subjects had lower levels of FT3 and FT4. There was also a relationship between the levels of FT3 and C-reactive protein (CRP) in the non-surviving group: the higher the CRP level, the lower the patient’s serum FT3 level. This indicates that the more severe the inflammation, the lower the body’s metabolic function, which is characterized by low FT3 levels [19].

In South Korea, Lee et al. documented the prevalence of NTIS in 213 ICU patients. They found that the incidence of moderate and severe NTIS was higher among infectious diseases patients compared to those without infectious diseases (39.8% moderate NTIS and 8% severe NTIS in patients with infectious diseases vs. 30.7% moderate NTIS and no severe NTIS in patients without infectious diseases). Patients with moderate or severe NTIS had a 3.1 times higher mortality risk than subjects with mild NTIS or no NTIS. Additionally, there was an increase in CRP levels with worsening NTIS, such that higher CRP levels were related with lower T3 levels (r = −0.199, p = 0.004) [20].

Lui et al. performed a study on 367 Coronavirus disease 2019 (Covid-19) patients in Hong Kong, China, who were treated during the pandemic. Compared to those who did not, individuals who underwent clinical deterioration had lower TSH and FT3 levels (median TSH: 0.85 vs. 1.25 mIU/L; median FT3: 3.6 vs. 4.2 pmol/L). Patients with NTIS were 3.19 times more likely to experience clinical worsening than those without NTIS [21].

Table 2 presents a summary of the relationship between thyroid hormones, NTIS, and outcomes in sepsis and infection patients.

**Table 2.** The association between NTIS, thyroid hormones and outcomes in patients with infection and sepsis

**Authors**	**Country**	**Subjects**	**Age**	**Main Findings**
Yao et al.[17]	Shanghai, China	1219 septic patients, 318 with DIC, 831 without DIC	70 years (mean)	Sepsis patients with NTIS had 3.19 times higher risk to suffer DIC. The higher the DIC score the higher NTIS prevalence

Abdelgawad et al.[18]	Cairo, Egypt	40 children with sepsis and septic shock	6 to 120 months	NTI prevalence was lower in sepsis (30%) compared to septic shock (65.5%) patients.
				FT3 levels were higher in 5th day, rT3 levels were lower in 1st and 5th day in survive compared to non-survive patients

Sharma et al.[19]	New Delhi, India	40 neonates patients with sepsis, 40 control subjects	neonates	FT4 and FT3 levels were lower in septic patients, and also lower in non-survive compared to survive patients
				The higher the CRP levels the lower the FT3 levels in survive-septic shock and non-survive patients

Lee et al.[20]	Seoul, South Korea	213 ICU patients	mean 58.9 years (euthyroid group), 69.1 years (mild NTIS), 66.9 years (moderate NTIS), 64 years (severe NTIS)	Subjects with infection had higher prevalence of moderate (39.8% vs 30.7%) and severe NTIS (8% vs 0%) compared to non infection patients. Subjects with moderate to severe NTIS had 3.1 times higher mortality risk compared to normal-mild NTIS

## NTIS and its relationship to outcomes in non-infectious diseases

### Cardiovascular diseases

Patients with cardiovascular diseases, especially those with acute coronary syndrome (ACS), are also more likely to have NTIS. The frequency of NTIS was 48% in unstable angina patients, 54% in non-ST elevation myocardial infarction patients, and 56% in ST-elevation myocardial infarction patients, according to a research conducted in Malaysia on 85 ACS patients by Adawiyah et al. They also found that blood levels of FT3 were negatively associated with peak serum troponin T levels (r = −0.22, p = 0.049), illustrating that FT3 levels are associated to the extent of myocardial infarction [22]. Dal and Topacoglu, in Turkey, reported on a study of 70 patients with myocardial infarction. They found that FT3 levels were lower in non-survived patients during treatment than in survived ones (2.0±0.1 pmol/L vs. 2.6±0.5 pmol/L) [23].

Zhang et al. in Shanghai, China, conducted a study of 501 patients who had experienced an acute myocardial infarction. The researchers conducted a one-year follow-up to assess mortality and major adverse cardiac events (MACE) frequency. The researchers found higher mortality in low FT3 patients compared to those with normal FT3 levels (14% vs. 2.7%), as well as an increased frequency of MACE during the period of one-year follow-up (66.7% vs. 45.5%). FT3 acted as a predictor of both mortality (hazard ratio [HR] = 0.142, p < 0.001) and MACE (HR = 0.748, p = 0.007) during that period [24].

NTIS is also found in heart failure patients and is associated with worse outcomes and higher mortality. Okayama et al. in Tokyo, Japan, studied 270 patients admitted with acute decompensated heart failure (ADHF) and revealed that admission FT3 levels were higher in survivors than in nonsurvivors (median 2.37 pg/mL vs. 1.65 pg/mL). An FT3 level below 2.05 pg/mL can predict mortality during treatment, with an AUC value of 0.791 (specificity = 72%, sensitivity = 85%). Subjects with low FT3 had a 9.62 times higher risk of in-hospital cardiac death compared to subjects with normal FT3 levels. In contrast to 14.2% of patients with normal FT3 levels, 29.9% of patients with low FT3 died during the one-year follow-up period (Kaplan-Meier survival curve, p < 0.001) [25]. In China, Zhao et al. studied 594 euthyroid individuals (with normal TSH and FT4 values) who were hospitalized with ADHF. Subjects were separated into two categories: those with or without low FT3. The researchers found that, although FT3 was not associated with in-hospital mortality (HR = 1.58, p = 0.290), FT3 was linked to mortality during one-year surveillance (HR = 1.85, p = 0.005) [26]. It’s interesting to note that Hayashi et al. in Japan reported a somewhat different finding. They studied 274 ADHF patients and discovered that low T3 was not a significant predictor of cardiovascular events (HR = 1.05, p = 0.858) and that subclinical hypothyroidism was a predictor of cardiovascular events (HR = 2.31, p<0.001). According to the authors, scheduling variations in thyroid function tests might be the cause of the disparity between their results and those of other reports [27].

### Kidney diseases

NTIS is also found in various kidney disease conditions. Obasuyi and Emokpae reported that 42.4% of 184 chronic kidney disease (CKD) patients in Nigeria had NTIS, primarily in advanced stages of CKD (stage 3: 54.5%; stage 4: 51.1%; stage 5: 37.2%). CKD patients had lower thyroid hormone parameter values than control subjects (TSH = 1.58 ± 0.09 vs. 3.05 ± 0.08 μIU/mL, p < 0.0001; T4 = 5.72 ± 0.14 vs. 10.2 ± 0.31 μg/dL, p < 0.001; T3 = 0.72 ± 0.03 vs. 1.34 ± 0.09 ng/dL, p < 0.001; and FT3 = 1.74 ± 0.07 vs. 2.51 ± 0.13 pg/mL, p < 0.001) [28]. Yuasa et al. in Japan, reported that 169 (33.1%) of 510 CKD patients had NTIS. Urine protein level (OR = 1.14, p < 0.001), estimated glomerular filtration rate (eGFR) (OR = 0.97, p = 0.047), and age (OR = 1.03, p = 0.068) were variables linked to the occurrence of NTIS in CKD patients [29]. Li et al. in China studied 317 patients with nephrotic syndrome and found that the incidence of NTIS (characterized by low T3 and or low T4) was high. The prevalence of NTIS in minimal-change disease was reported at 36.11%, in membrane proliferative glomerulonephritis at 42.86%, in mesangial proliferative glomerulonephritis at 66.66%, in membranous nephropathy at 31%, in focal segmental glomerulonephritis at 45.45%, in IgA nephropathy at 34.61%, and in secondary causal nephrotic syndrome at 38%. High levels of serum creatinine, cholesterol, platelets, and urinary protein, as well as low levels of blood hemoglobin and albumin, are predictors of thyroid dysfunction in nephrotic syndrome subjects [30].

### Other diseases (pulmonary, liver, diabetes mellitus, systemic lupus erythematosus (SLE), psychiatric disorders, sarcopenia)

NTIS is also found in a wide spectrum of diseases and is linked to clinical outcomes. Yasar et al. conducted a study in Turkey on 125 ICU patients with chronic obstructive pulmonary disease (COPD) who received mechanical ventilation and found a 53.8% prevalence of NTIS. Patients who experienced NTIS had higher APACHE II scores (median 29 vs. 24) than those who did not, indicating worse clinical conditions. Additionally, the NTIS group had lower levels of FT3 and FT4 than the non-NTIS group (median FT3 = 1.60 pg/mL vs. 2.48 pg/mL; median FT4 = 0.97 pg/mL vs. 1.21 pg/mL). NTIS was also a predictor of prolonged mechanical ventilation (OR = 3.21, p < 0.001) [31].

Langer et al. studied 437 patients with hepatic disorders in Germany and found that NTIS occurred in 72.1% of patients with acute-on-chronic liver failure (ACLF) and 39.9% acute decompensated liver failure (AD) patients. Subjects with ACLF had the lowest FT3 levels, followed by subjects with AD and then subjects with compensated heart failure (mean FT3 levels: 3.1, 3.7, and 4.8 pmol/L, respectively, p < 0.001). A Cox regression test showed that low serum FT3 levels were linked to mortality during the 3-month monitoring period (OR = 0.71, p = 0.002) [32].

In Guangzhou, China, Deng et al. studied 396 adults hospitalized with diabetic ketoacidosis (DKA) or diabetic ketosis (DK) and found an NTIS prevalence of 57.8%. The prevalence of DKA was higher in the NTIS subjects than in the non-NTIS ones (27.9% vs. 18%), as were the incidences of acute kidney injury (21.7% vs. 11.6%) and co-infection (67% vs. 27.4%). FT3 was negatively associated with high-sensitivity C-reactive protein (hsCRP) (r = −0.440, p < 0.001), urinary albumin (r = −0.15, p < 0.001), and leukocyte count (r = −0.504, p < 0.001) and positively correlated with acidosis parameters, such as partial pressure of carbon dioxide (PaCO_2_) (r = 0.309, p < 0.001) and hydrogen carbonate (HCO_3_^−^) (r = 0.285, p < 0.001), among patients with DKA and DK [33].

Zhang et al. in Nanjing, China conducted a study on 223 subjects with SLE and found SLE subjects with NTIS had higher SLE disease activity index (SLEDAI) values than non-NTIS (mean 8.5±4.6 vs 4.0±3.6), as well as blood creatinine levels, blood urea nitrogen, uric acid, CRP, and erythrocyte sedimentation rate (ESR) [34].

Sakai et al. conducted a study in Norway on adult patients with acute psychiatric disorders who were receiving psychiatric care. The researchers generally found increased TSH and FT4 levels in acute psychiatric disorders subjects compared to reference values in the general population. Serum FT4 levels above the reference range (>19.1 pmol/L) were found in 15.7% of patients with neurotic disorders, 16% with substance use disorder (SUD), 20.6% with bipolar depression, 21.7% with unipolar depression, 23.5% with personality disorders, 26.1% with schizophrenia, and 31.2% with mania. Increased TSH levels above the reference range (>3.78 mIU/L) were found in 2% of patients with schizophrenia, 2.6% with unipolar depression, 3.6% with SUD, 3.8% with neurotic disorders, 6.2% with mania, 11.8% with bipolar depression, and 15.2% with personality disorders [9].

Chen et al. in Nanjing, China, conducted a study on 309 elderly subjects (the vast majority of them over 80 years old) and found that T3 and FT3 levels were significantly lower in subjects with sarcopenia than in those without. Low T3 levels are associated with reduced muscle strength in elderly subjects. Normal T3 levels act as a protective factor against the development of sarcopenia in this population [35].

Table 3 summarizes the connection between thyroid hormones, NTI, and outcomes in non-infectious illnesses patients.

**Table 3.** The relationship between NTI, thyroid hormones and outcomes in patients with non-infectious diseases

**Authors**	**Country**	**Subjects**	**Age**	**Main Findings**
**Cardiovasular Disease**				

Adawiyah et al.[22]	Kuala Lumpur, Malaysia	85 ACS patients, 6-month cohort study	58.3 years (mean)	NTIS prevalence was 56% in ST elevation myocardial infarction, 54% in non-ST elevation myocardial infarction, and 48% in unstable angina
				Peak Troponin T levels was associated with FT3 levels (r =−0.22, p = 0.049)

Dal et al.[23]	Istanbul, Turkey	70 acute myocardial infarction patients	64.46 years (mean)	Non-survive acute myocardial infarction patients had lower FT3 levels compared to survive ones

Zhang et al.[24]	Shanghai, China	501 acute myocardial infarction patients, cohort study (mean follow up 10±2 months)	69 years (mean)	Mortality rate in low FT3 subjects were higher than normal FT3 (14 vs 2.7%). FT3 level was determinant factor of mortality and MACE (HR = 0.142 and 0.748, p<0.05 respectively) during follow up

Okayama et al.[25]	Tokyo, Japan	270 ADHF patients	mean 65 years (normal FT3 group) and 72.3 years (low FT3 group)	FT3 (cut-off <2.05 pg/mL) has the largest AUC (0.791) to predict mortality risk (specificity = 0.72, sensitivity = 0.85)

Zhao et al.[26]	Gansu, China	594 ADHF patients with euthyroid	57 years (mean)	Low FT3 level was not associated with mortality during hospitalization but associated with 1 year mortality (HR = 1.85)

Hayashi et al.[27]	Japan	274 ADHF patients	70 years (mean)	Subclinical hypothyroidism (HR = 2.31) but not low FT3, associated with cardiovascular events

**Kidney Disease**				

Obasuyi et al.[28]	Benin, Nigeria	184 CKD subjects, 80 controls	5 to 90 years	42.4% CKD patients had NTIS (54.5% of stage 3; 51.1% of stage 4; and 37.2% of stage 5 CKD patients)

Yuasa et al.[29]	Tokyo, Japan	510 CKD subjects	67 years (median)	33.1% of CKD patients had NTIS. Urine protein, eGFR, and age were determeninat factor of NTIS

Li et al.[30]	Chengdu, China	384 nephrotic syndrome patients	41.3 years (mean, normal thyroid subjects); 40.65 years (mean, thyroid dysfunction subjects)	NTIS prevalence ranged from 31% to 66.66% among different nephrotic syndrome types. Blood creatinine, cholesterol, platelet, hemoglobin, albumine, and urine protein are determinant factors of thyroid dysfunction

**Other Diseases**				

Yasar et al.[31]	Turkiye	125 COPD subjects	65 years (mean)	NTIS prevalence in COPD patients was 53.8%. NTIS was linked to higher APACHE II scores (29 in NTIS vs 24 in non-NTIS)

Langer et al.[32]	Germany	437 liver cirrhosis patients	52.1 to 55.8 years (mean, among liver cirrhosis type)	NTIS prevalence was 72.1% in ACLF and 39.3% in AD patients. Subjects with low FT3 had higher 3-month mortality rate

Deng et al.[33]	Guangzhou, China	396 diabetic ketoasidosis and ketosis patients	57.5 years (mean)	NTIS prevalence was 57.8% among diabetic ketoasidosis and ketosis patients. FT3 had negative association with blood hsCRP, urine albumine levels, and leukocytes count

Zhang et al.[34]	Nanjing, China	223 SLE patients	36.7 years (mean, subjects with NTIS); 36.9 years (subjects without NTIS)	Prevalence NTIS in SLE patients was 58.74%. FT3 had negative association with SLE activity (r =−0.313), creatinine (r =−0.298), blood urea nitrogen (r =−0.325), CRP (r =−0.200) and urine protein (r =−0.301) among NTIS patients

## Discussion

Thyroid hormones (T3 and T4) are synthetized by the thyroid gland. The thyroid hormones exert their effects on various target organs through the action of iodothyronine deiodinases, enzymes that are expressed by different tissues. The iodothyronine deiodinases (DIO) enzymes are responsible for several functions, including the transformation of T4 to biologically active T3 (by isoenzymes DIO1 and DIO2), the conversion of T4 to biologically inactive rT3 (by isoenzymes DIO1 and DIO3), the conversion of T3 to diiodothyronine (T2) (by isoenzymes DIO1 and DIO3), and the conversion of rT3 to T2 (by isoenzymes DIO1 and DIO2) [7].

In patients diagnosed with NTIS, the predominant finding on their thyroid hormone panel is reduce of T3 levels and elevate of blood rT3 levels. Decreased blood T3 levels have been observed in mild NTIS. In mild NTIS, TSH and FT4 levels are initially within normal limits. However, if the disease condition is prolonged or worsens, it will usually be shown by gradual reduction in TSH, FT4, and T4 levels. As the disease progresses and the treatment period extends, there is a concomitant decrease in T3 levels and an increase in rT3 levels. Decreased TSH, T3, and T4 levels in NTIS, along with increased rT3 levels, have been observed to be related with higher mortality risk. If the patient recovers, TSH levels will return to normal and may even temporarily increase above standard reference values. Concurrently, T3 and T4 levels will gradually revert to normal, while rT3 levels will decrease to within normal parameters [7].

A variety of acute medical conditions have been demonstrated to induce NTIS, including, but not limited to, respiratory ailments, cardiovascular diseases, acute infections, trauma, and burns. During the initial stages of critical illness, there is a transient increase in TSH and T4 accompanied by a fall in T3 and a rise in rT3 levels [8]. In NTIS, there is impaired synthesis and increased degradation of thyroid hormone-binding proteins, such as transthyretin, albumin, and thyroxine-binding globulin (TBG), causing total T3 and T4 levels in the blood plasma to decrease [7]. Increased levels of functionally inactive rT3 are caused by increased expression of DIO3 enzymes in skeletal muscle and liver that convert T4 to rT3. Increased expression of DIO2 in macrophages has been observed to trigger a localized increase in T3 levels, which has the potential to enhance the macrophages’ ability to phagocytize and release inflammatory mediators, thereby amplifying their capacity to eradicate bacteria in infectious conditions. In critical conditions, hepatic DIO1 expression is reduced, leading to reduction of T4 to T3 conversion. These results suggest that alterations in thyroid hormones in the critical illness acute phase (comparable to central hypothyroidism) contribute to enhanced patient resilience against infection and the body’s regulatory efforts to optimize energy expenditure and reduce basal energy requirements, thereby promoting patient survival [8].

In patients with chronic or prolonged critical illness, thyroid test findings typically reveal a more pronounced decline in blood T3 levels compared to the acute phase. This decline is often accompanied by a reduction of blood TSH and T4 levels. In cases of chronic conditions, there is a decrease in hypothalamic TRH production, resulting in the suppression of TSH and T3 production. Continued suppression can have detrimental effects, including impaired organ function and impaired cognitive function due to thyroid hormone deficiency. These effects are associated with increased mortality. In the event that the underlying disease is successfully addressed (recovery), the thyroid hormone pattern will undergo a gradual return to normalcy. Concurrently, T3, T4, and TSH levels will gradually revert to normal ranges as rT3 levels return to baseline values [8].

Changes in thyroid hormone patterns (similar to central hypothyroidism) in the critical illness acute phase indicate the body’s adaptation efforts to regulate optimal energy use and reduce basal body energy use in order to improve patient survival. In the event of a successful resolution of the underlying disease, the thyroid hormone pattern will undergo a gradual return to normalcy. However, in the case of persistent critical illness that evolves into a chronic state, the alterations in thyroid hormone pattern will persist, potentially inflict harm upon the body due to the onset of impaired function in various organs that are the target of thyroid hormone action and result in an elevated mortality rate [8].

Altered thyroid hormone patterns have also been observed in acute psychiatric patients. These patients frequently exhibit elevated FT4, T4, and TSH levels, indicative of central hyperthyroidism. Following the administration of therapeutic interventions and subsequent discharge from the treatment center, the majority of patients exhibit a decline in FT4 levels, despite a relatively stable TSH level [9].

Thyroid hormone patterns changes in NTIS are summarized in Table 4.

**Table 4.** Changes in thyroid hormone patterns in NTIS

**Thyroid Hormones**	**Acute Phase of Critical Illness**	**Chronic Phase of Critical Illness**	**Recovery Phase of Critical Illness**
TSH	N or slightly ↓	N or slightly ↓	N or slightly ↑
FT4	N or slightly ↑	N or slightly ↑	mostly N
FT3	N or gradual ↓	↓↓	gradual ↑ to baseline
T3	N or gradual ↓	↓↓	gradual ↑ to baseline
T4	N or slightly ↓	↓↓	gradual ↑ to baseline
rT3	N to gradual ↑	↑↑	gradual ↓ to baseline

## Conclusions

An increased incidence of NTIS in patients with critical illness treated in the ICU is linked to poor outcomes and increased mortality. A complete thyroid function test panel is needed to examine critically ill patients at the beginning of admission and during treatment to assess the occurrence and severity of NTIS, as well as deterioration and recovery from the disease. Early diagnosis of NTIS is crucial, primarily because thyroid hormone disorders are associated with the severity and prognosis of underlying diseases, including a significant risk of mortalities in critically ill patients. Detecting NTIS is also necessary for assessing the outcome and prognosis of various diseases, both infectious and non-infectious, during treatment, short and long-term monitoring. Prompt identification of NTIS can provide an overview of the severity of the patient’s clinical condition, enabling appropriate medical intervention to reduce morbidity and mortality.
